# Eccentric exercise is more effective than other exercises in the treatment of mid-portion Achilles tendinopathy: systematic review and meta-analysis

**DOI:** 10.1186/s13102-023-00618-2

**Published:** 2023-01-26

**Authors:** Diego Ailton Prudêncio, Nicola Maffulli, Filippo Migliorini, Thiago Teixeira Serafim, Luis Felipe Nunes, Luciana Sayuri Sanada, Rodrigo Okubo

**Affiliations:** 1 Department of Physiotherapy, Physiotherapy Postgraduation Program (PPGF), Santa Catarina State University, Florianópolis, Brazil; 2 Department of Orthopaedics, School of Medicine, Surgery and Dentistry, University of Salerno, Salerno, Italy; 3 School of Pharmacy and Bioengineering, Faculty of Medicine, Keele University, Stoke On Trent, UK; 4 Centre for Sports and Exercise Medicine at Queen, Mary University of London, London, UK; 5 Department of Orthopaedic, Trauma, and Reconstructive Surgery, RWTH University Hospital, Pauwelsstraße 30, 52074 Aachen, Germany; 6 Department of Pharmacy, Federal University of Santa Catarina, Florianópolis, Brazil

**Keywords:** Ankle injuries, Achilles tendinopathy, Review, Physiotherapy modalities, Exercise therapy

## Abstract

Achilles tendinopathy (AT) is one of the most frequent overuse injuries in the ankle. The evidence base for its conservative management AT continues to evolve, but there is still a gap in the evidence for the efficacy of any modality of treatment in high-quality studies. This systematic review and meta-analysis investigated the efficacy of EE in improving pain and function in adult patients with mid-portion Achilles tendinopathy compared to other forms of exercise. A search was performed in PubMed, BIREME, SportDiscus, Cinahl, Web of Science and PEDro, in November 2022. The methodological quality was evaluated using the Risk of Bias 2 tool (RoB2) of the Cochrane collaboration, and the meta-analysis was performed using the Review Manager 5.1 program. 2024 articles were identified and eight fulfilled the inclusion criteria. RoB2 presented a final score with 62.5% of the studies presented “some concerns”, and 37.5% (five and three articles, respectively) presenting “high risk” of bias. EE was effective for the managment of AT. The only variable for which a meta-analysis was possible was pain (five articles), analysed with the visual analogue scale/numerical visual scale. The mean difference (MD) in treatment effect using EE was − 1.21 (− 2.72 to − 0.30) with a 95% of confidence interval (CI), thus identifying a significant positive effect for the improvement of pain in patients with AT in whom EE was used. EE is effective in the management of AT. The meta-analysis shows the need for appropriately powered randomized controlled trials with better design, the use of standard outcome measures and well-planned protocols for conservative management of AT.

*Level of evidence*: Level 1.

*Registration*: CRD42018118016.

## Introduction

The Achilles tendon is one of the widest and strongest tendons in the human body. Despite this, injuries are common [1]. Achilles tendinopathy (AT) is one of the most frequent overuse injuries in the ankle and foot [2–4], and is a clinical syndrome characterized by pain, swelling and loss of function [5].

AT has an incidence of 1.85 per 1000 people in the general population [5, 6]. Most individuals with AT are active, and involved in recreational or competitive sports [7, 8]. For example, runners have an annual incidence of 10%, with a higher chance of developing AT symptoms compared to non-runners younger than 35 years [7, 9]. AT can be present in 51/100 of athletes in whom running is part of their sport [8].

Basketball, soccer, tennis and wrestling have high incidence rates of ankle injuries [10, 11]. Up to 27% of all musculoskeletal injuries in athletes' are foot and ankle injuries, with the highest incidence in female gymnastics, female soccer and male and female cross-country runners [12]. However, up to one in three sedentary individuals can develop AT [6].

Abnormal biomechanics of the lower limb may increase the risk of excessive or unusual loading of the Achilles tendon, and changes in range of motion (ROM) of the ankle and lower limb have been associated with an increased risk of AT [13]. In most AT patients, the condition improves with simple conservative interventions, the first line management option, which can be combined with a variety of other agents [14–16]. Surgery is generally undertaken when appropriately conducted conservative management has failed [17].

Conservative therapies for the management of AT include kinesiotherapy, electrotherapies, cryotherapy, dry needling, bandaging, splints[18–21]. Exercise programs are the most widely studied interventions for the management of AT, and eccentric, concentric, isometric, and isokinetic contractions have all been used, alone or in combination [15, 22–26]. In 2012, a systematic review with a meta-analysis [23] in relation to physical therapy in AT identified 23 publications: eccentric exercise (EE) was shown to be an effective intervention to manage AT [23]. More recently, other studies have shown the efficacy of EE for the management of AT [15, 16, 27–29].

In eccentric exercise, the muscle contracts while being lengthened [30]. Eccentric contractions not only produce the highest forces in the muscle compared to concentric or isometric contractions, but are also energy efficient [31]. The most frequently described protocol focuses on a progressive eccentric strengthening with increasingly heavier loads [32]. Van der plas et al. [33] evaluated EE for 3 months at 5 years of follow-up: EE is effective in increasing function and controlling pain symptoms. Roos et al. [34] concluded that EE reduces pain and improves function in AT patients, and more patients in the EE group returned to their sport after 12 weeks than the other groups.

The prognosis of AT may vary from individual to individual. Paavola et al., in a 8-year follow-up study, showed that 84% of the patients returned to their pre-disease activity levels, and 94% of the patients were asymptomatic or had only minimal pain [17].

The evidence base for conservative therapy for AT continues to evolve, but there is still a gap in the evidence for its effectiveness in high-quality studies. We performed a systematic review and meta-analysis to investigate the efficacy of EE in improving pain and function in adult patients with mid-portion Achilles tendinopathy compared to other forms of exercise.

## Methods

The protocol for this review was registered in the International prospective register of systematic reviews—PROSPERO, number CRD42018118016, the PRISMA guidelines (Preferred Reporting Items for Systematic Reviews and Meta-Analyses) and the AMSTAR 2 (A MeaSurement Tool to Assess systematic Reviews) were used.

### Searches

The search was performed in PubMed, BIREME, SportDiscus, Cinahl, Web of Science and PEDro without date restriction filter. Table 1 shows the search strategy relationship, which was adapted for use in other databases.

**Table 1:** Keywords of the search strategy of the electronic databases

Search	PubMed/sportdiscus/cinahl/web of Science	PEDro	BIREME
#1	Achill*(.tw)	achill*	Achill*
#2	Triceps surae(.tw)	tendin*	Triceps surae
#3	Tendin*(.tw)	eccentric*	Tendin*
#4	Heel(/)	concentric*	Heel
#5	1 OR 2 OR 3 OR 4	exercise	Pain
#6	Pain(/)		Function
#7	Function(.tw)		Physical therapy
#8	6 OR 7		Physiotherapy
#9	5 AND 8		Exercise therapy
#10	Physical therapy(.tw)		Exercise
#11	Physiotherapy(.tw)		Rehabilitation
#12	Exercise therapy(.tw)		Concentric*
#13	Exercise(/)		Eccentric*
#14	Rehabilitation(/)		Strength training
#15	Concentric*(.tw)		Strengthening
#16	Eccentric*(.tw)		Resistance training
#17	Strength training(.tw)		Randomized controlled trial
#18	Strengthening(.tw)		Controlled clinical trial
#19	Resistance training(/)		Randomized controlled trials as topic
#20	10 OR 10 OR 12 OR 13 OR 14 OR 15 OR 16 OR 17 OR 18 OR 19		Trial
#21	Randomized controlled trial(/)		Placebo
#22	Controlled clinical trial(/)		
#23	Randomized controlled trials as topic/		
#24	Trial(.tw)		
#25	Placebo(.tw)		
#26	21 OR 22 OR 23 OR 24 OR 25		
#27	9 AND 20 AND 26	1 ADN 2 AND 3 AND 4 AND 5	1 AND 2 AND 3 AND 4 AND 5 AND 6 AND 7 AND 8 AND 9 AND 10 AND 11 AND 12 13 AND 14 AND 15 AND 16 AND 17 AND 18 AND 19 AND 20

*Truncation (search term starting with the letters preceding the asterisk)

.tw, text word; /, Mes

Following identification of the articles which matched the inclusion and exclusion criteria, their references were manually searched to identify other possible articles to be included in the present study.

### Study inclusion and exclusion criteria

As randomized clinical trials (RCT) are the gold standard to assess the effectiveness of clinical research [35], we included RCT with eccentric exercise performed in adult patients (older than 18 years) with mid-portion Achilles tendinopathy in whom a programme of eccentric exercises was compared to another conservative modality, and in whom the outcome measure was assessment of pain and/or disability. Regarding the studies selection, we were interested in identifying the effects and harms associated with an intervention. Hence, RCTs were chosen because they can provide a complete overview of the efficacy of a given intervention.

We did not include studies reporting on participants with ruptured Achilles tendon or with insertional tendinopathy. The search was limited to human studies and published in Portuguese, English or Spanish, due to the language capabilities of the researchers. In addition, review articles, expanded abstract, letters to the editor, annals of congress, editorials, dissertations and theses were excluded.

### Data extraction

The following information was extracted: basic information (author, published year, country, trial design, subject characteristic, sample size, intervention duration, basic results); and outcome measures (primary—pain and function; secondary—strength and range of movement). The search was performed by two researchers independent of each other. If there was disagreement, a third reviewer was consulted for final decision. Data were exported and stored in the Zotero® program, where duplicate articles were excluded. The data were then exported to Microsoft Excel®, where articles were removed according to their titles, followed by their abstract, and then the remaining articles were read in full text. Relevant data were then extracted manually and inserted into Review Manager software (RevMan 5.3).

### Study quality assessment

The Cochrane Collaboration risk-of-bias 2 (RoB 2) tool for randomized trials was used to assess the risk of bias in the included studies [36]. The tool is structured into five domains through which bias can be introduced. The domains cover all types of trends that may affect the results of randomized trials namely: (1) bias arising from the randomization process; (2) bias from deviations from intended interventions; (3) bias from missing outcome data; (4) bias in measurement of the outcome; (5) bias in selection of the reported result. The answer options for the questions are: yes; probably yes; probably no; no; and no information. Responses to questions provide the basis for domain-level judgments about risk of bias, and then these domain-level judgments provide the basis for a general risk of bias judgment for the outcome of the study being evaluated. The possible judgments of risk of bias are: (1) low risk of bias; (2) some concerns; and (3) high risk of bias [36].

### Data synthesis and presentation

A meta-analysis was performed to synthesize study data and verify the effect size of the intervention using the RevMan 5.3 program. The results of the meta-analyses are presented in a forest plot, where the left side positively represents the treatment (less than zero) and the right side negatively (greater than zero). We used for the meta-analysis an effect model with a justification of the same AT population, and a mean difference as the type of effect size. Each study is shown with its effect size and the corresponding 95% confidence interval [37, 38]. The general measure of the effect is represented by a diamond: its centre represents the overall estimate, and the width or side points indicate general confidence intervals [39]. Heterogeneity was tested by *p* value and I2. If *p* > 0.10 and I^2^ < 50%, the heterogeneity was considered low enough to conduct a meta-analysis with a fixed-effect model. If *p* < 0.10, I^2^ > 50%, there was a high level of heterogeneity, and a random effect model was used. Sensitivity analysis was conducted by one-by-one exclusion method for individual studies. Descriptive analysis was performed if the heterogeneity was too large (I^2^ > 85%).

## Results

### Search results

**Fig. 1:**
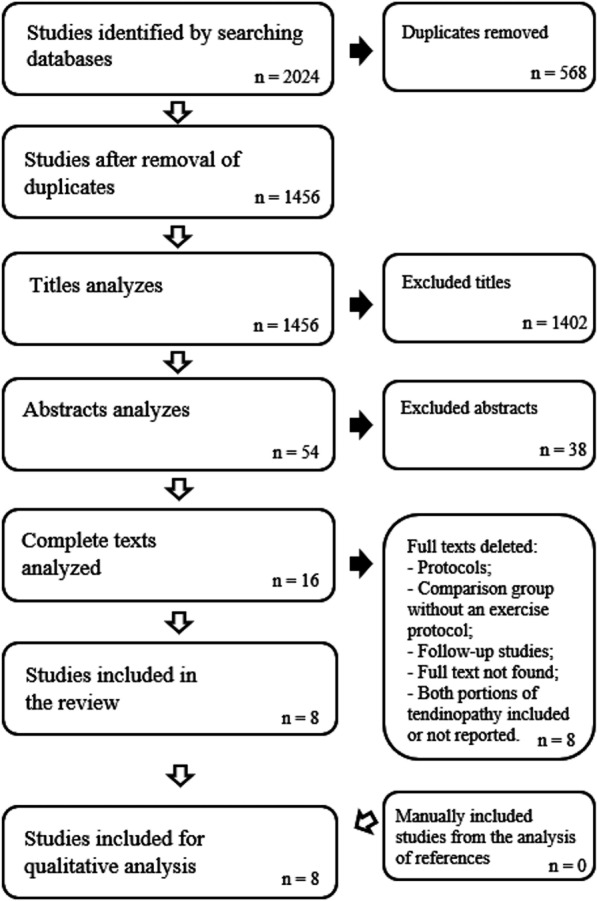
Flow chart of the literature search

A total of 2024 articles were identified among the published articles to the time when the search was performed, with the last update in November 2022. After all stages of the review, 16 articles remained for complete analysis. Studies were excluded after the full-text screening for the following reasons: protocols [40]; comparison group without an exercise protocol [34, 41–43]; follow-up studies [33, 44]; full text not found [45]; both tendinopathy of the main body and insertional tendinopathy included in the study or not reported [46, 47]. Therefore, eight articles fulfilled the inclusion criteria. No article was identified through the manual search in the references section. A total of 8 studies were eventually included in the present study (Fig. 1). The studies were conducted in seven different countries: Sweden [48–50] and Germany [51, 52] with one each in Greece [53], Scotland [54] and Denmark [55] respectively.

### Participants

The included studies reported a total of 401 Achilles tendons in 371 patients; two studies included individuals with bilateral and unilateral AT [49, 50]. Clinical examination (7/8) was the most common criterion for the diagnosis of AT [48–50, 52–55]; three studies used ultrasound [48, 51, 55], and one [55] added the VISA-A questionnaire and the VAS pain scale to complete the diagnosis. All studies included only patients with tendinopathy of the main body of the Achilles tendon. The minimum duration of symptoms was of three months. The participants' ages ranged from 19 to 77, with a mean of 46.6 years. Male participants were 54.2%, and 45.8% were females in the articles that reported the gender, with one article [53] not reporting the sex of the participants.

The samples per study group ranged from 13 to 25 participants, with a mean of 18.5 individuals in each group.

### Interventions

All studies were clinical trials. Six studies included two intervention groups: one was eccentric exercise (EE), compared to another exercise modality, such as concentric exercise [48]; light training [49]; heavy slow resistance (HSR) [55]; and with another protocol of eccentric exercise [50, 53, 54]. Two articles included three groups: EE, electrotherapy and wait-and-see [51] and EE, vibration training and wait-and-see [52]. Most studies (75%) applied the intervention for 12 weeks, but Silbernagel et al. [50] extended the intervention to six months if symptoms persisted. Stevens et al. [54] undertook the intervention for 6 weeks. In addition, all articles evaluated change in pain; seven verified function/disability [49–55]; and three, patient satisfaction with treatment [48, 54, 55].

### Comparison and outcome measure

Five of the eight studies used the VISA-A scale to assess pain and symptoms [50, 51, 53–55], two used functional tests (jump tests, toe-raise test, hop test, side-step test) [49, 50], one used isokinetic strength [52], and two assessed ROM [49, 50]. Only one study [53] also used the VISA-A to evaluate pain evolution. The other articles used the Visual Analogue Scale/Numerical Visual Scale (VAS/NVS). Most of the articles performed the evaluations at the beginning and at the end of the treatment, with one study undertaking follow-up at 36 weeks [53], one at 6 months [49], and three at 1-year of follow-up [50, 51, 55]. Four studies showed better outcome in the EE treatment group [48, 49, 51, 52], one article did not present statistically significant differences [55]. Among the three articles comparing two eccentric exercises protocols, one study presented better results with the Alfredson protocol [53], and two studies did not find statistically significant differences between groups [50, 54] (Table 2).

**Table 2:** *Source*: Own author/2019

Study	Intervention(s)	Sample size	Gender (M/F)	Age (mean)	Intervention duration (wk)	Comparison and outcome measure	Study conclusions (*p* value/SMD—95% CI)
*Eccentric exercise vs another exercise therapy or rest/wait-and-see*							
Mafi 2001	EE	22	12/10	48.1 ± 9.5	12	VAS	The results after treatment with EE was significantly better (*p* < 0.002) than the results of treatment with the concentric training regimen; Between groups comparisons of pain not presented
	Concentric exercise	22	12/10	48.4 ± 8.3			
Silbernagel 2001	Eccentric overload training;	22	17/5	47 ± 14.7	12	VAS Jumping test; toe-raise test	Eccentric loading had better strength and pain outcomes (*p* < 0.05)
	Light training	18	14/4	41 ± 10.2			
Beyer 2015	EE	25	18/7	48 ± 2	12	VISA-A VAS_H_ VAS_R_	VISA-A: there was no significant interaction (*p* = 0.26) or difference between groups (*p* = 0.62). VAS_H_ and VAS_R_ there was no significant interaction (VAS_H_, *p* = 0.08; VAS_R_, *p* = 0.38) or difference between groups (VAS_H_ *p* = 0.77; VAS_R_, *p* = 0.71). Similar treatments
	HSR	22	14/8	48 ± 2			
Rompe 2007	EE	25	9/16	48.1 ± 9.9	12	1 vs 3 VISA-A VNE	VISA-A and VNE: Patients from group 1 achieved significantly better results than patients from group 3 (*p* < 0.001)
	SWT	25	11/14	51.2 ± 10.2			
	Wait-and-see	25	9/16	46.4 ± 11.4			
Horstmann 2013	Vibration training	23	13/10	46.0 ± 6.9	12	2 vs 3 VAS Isokinetic	Pain improvements were greater in the EE groups than in the wait-and-see group (– 27.0; 95% CI –50.9, – 3.1)
	EE	19	10/9	45.7 ± 8.5			
	Wait-and-see	16	9/7	44.4 ± 7.7			
Silbernagel 2007	EE	19	12/7	44 ± 8.8	6–52	VISA-A-S VAS Jump tests, strength tests, endurance test	Both groups showed significant (*p* < .01) improvements on the VISA-A-S score and decrease in pain during hopping at 6 weeks and at 3, 6, and 12-month evaluations
	EE + active	19	8/11	48 ± 6.8			
Stasinopoulos 2013	Stanish protocol	21	NR	48.44 ± 5.12	12	VISA-A	VISA- A: there were significant differences in the magnitude of improvement between the groups at weeks 12 and 36 (*p* < 0.05)
	Alfredson protocol	20		48.24 ± 5.09			
Stevens 2014	Alfredson protocol	15	6/9	48.2 ± 10.8	6	VISA-A VAS	The between-group difference change score was not statistically significant at week 6 for VISA-A (ITT, *p* = 0.20; PP, *p* = 0.32) and VAS (ITT, *p* = 0.14; PP, *p* = 0.73)
	Alfredson protocol (do-as-tolerated)	13	5/8	49.2 ± 11.3			

SMD, standard mean difference; M, male; F, female; NR, not reported; CI, confidence interval; EE, eccentric exercise; VAS, visual analogue scale; VISA-A, Victorian Institute of Sports Assessment-Achilles; HSR, heavy slow resistance; VAS_H_, visual analogue scale heel rises; VAS, VAS, visual analogue scale running; SWT, shock-wave therapy; VNE, visual numerical scale; VISA-A-S Victorian Institute of Sports Assessment-Achilles Swedish; ITT, intention-to-treat; PP, per-protocol

### Risk of bias

**Fig. 2:**
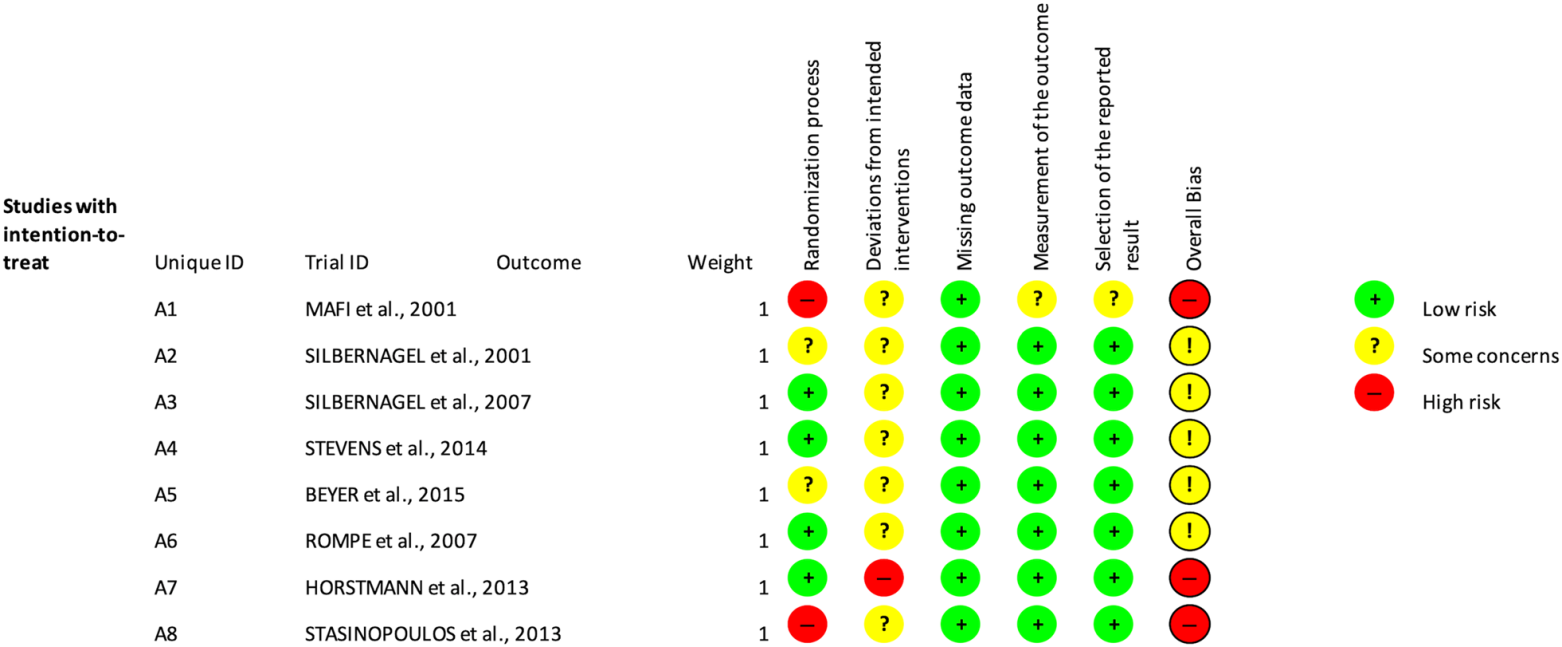
Variability of articles in relation to fundamental methodological considerations

**Fig. 3:**
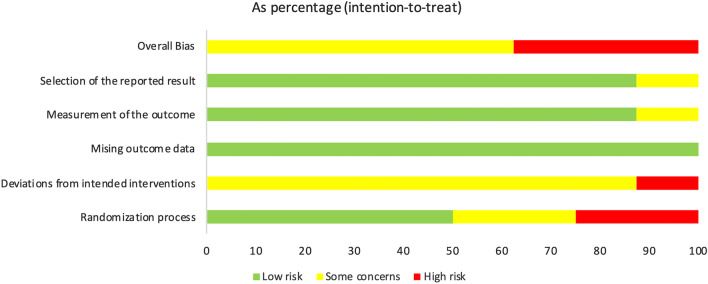
Methodological quality summary: analysis of the authors' judgments about each item of methodological quality for each included study

Figures 2 and 3 highlight the variability between the articles included and show the general score about the methodological quality. Figure 2 shows the risk of bias for each article in each domain analysed by the program. Figure 3 shows a general comparison between low risk, some concerns and high risk in each domain.

### Allocation

Regarding allocation of participants, 50% of the studies presented “some concerns” or “high risk” as they did not report details about how randomization and/or allocation to the various groups were performed [48, 49, 53, 55]. Four articles had adequate allocation concealment, including central randomization methods [50–52, 54].

### Blinding

No studies reported blinding of the participants, health care professionals who administered the treatment, and evaluators, presenting a low-risk score. One study did not present information about participants and health care professionals who administered the treatment [49]; the patients were aware of the treatment or no information was presented in four studies [48, 50, 53, 54]; the health care professionals who administered the treatment knew or probably knew about the treatments in six articles [48, 50–53, 55]. All except of one of them had blind evaluators [48].

### Missing results data

All the articles presented the missing data reporting the reasons of exit from the study or exclusion from the intervention, being judged with low risk of bias.

### Evaluation of results

Seven of the eight studies presented evaluation of appropriate results, with “low risk”. Only one study presented “some concerns” [48] for non-blinding the evaluators. None of the selected articles showed “high risk” of bias.

### Selection of the reported result

Only two articles did not provide evidence of previous protocols published in study databases [49, 52], and one article presented only data for the intervention group [48]. This one study, however, did not present the statistical analysis performed, being thus judged with “some concern”.

### Overall bias

Regarding the final score of risk of bias assessment, 62.5% of the studies presented “some concerns”, and 37.5% presented “high risk” of bias.

### Meta-analysis

**Fig. 4:**
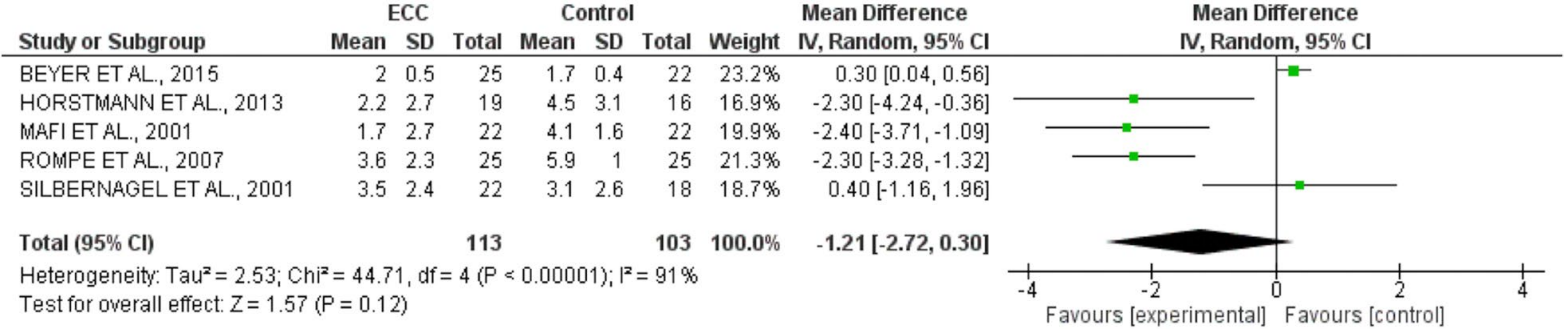
Meta-analysis for pain intervention with EE

Only a meta-analysis with pain data was performed including a total of five studies. All of them used VAS/NVS to measure pain, and compared the EE with another conservative treatment (Fig. 4). The analysis of heterogeneity resulted in the I^2^ value of 91%, showing heterogeneity between the studies. A meta-analysis with I^2^ higher than 75% should use the Random Effect Model. The Mean Difference (MD) found in the treatment effect was − 1.21 (− 2.72 to − 0.30), with 95% confidence interval, with a significant positive effect for the treatment of pain in Achilles tendinopathy with eccentric exercise, with the result in favour of EE compared to CG or other exercises in pain improvement.

## Discussion

The present systematic review and meta-analysis investigated the efficacy of EE in improving pain and function in adult patients with mid-portion Achilles tendinopathy compared to other forms of exercises. The present systematic review and meta-analysis showed that EE is effective in improving disability and pain in AT patients. In the eight studies evaluating the effect of pain and disability of EE with another type of exercise, four presented favourable EE results for pain [48, 49, 51, 52], and one presented similar results between interventions [55]. Regarding the effect on disability, two articles were in favour of EE [49, 51].

The Victorian Institute of Sport Assessment-Achilles questionnaire (VISA-A) was the most commonly used outcome instrument (used in five of eight investigations) in the studies assessing disability. This questionnaire is used as an outcome measure in both research studies and in the clinical setting, and it evaluates function, activity limitation or participation restriction, in addition to documenting pain and stiffness [15]. Two studies used functional tests, the hop and heel-raise endurance tests being the most common. These tests should be used as part of the evaluation, are useful to assess progress during rehabilitation, and are often used to detect changes over time [15, 56]. Among the studies that evaluated pain, the visual analogue scale/numerical visual scale were the most commonly used (seven of eight). One study [53] used the VISA-A questionnaire to evaluate pain and function. Visual scale values can be used to track the progression of pain in a single patient, or to compare pain among patients with similar conditions [57]. While there is conflicting evidence regarding the advantage of EVA/NVS compared to other pain recording methods, it is still commonly used in clinical and home settings [58, 59].

EEs improve muscle function, possibly through their favourable effects on neural impulse and other health-related factors, and do not produce clinically relevant adverse effects [60–62]. EE may promote the formation of appropriately oriented collagen fibers in the tendon, which may facilitate remodelling of the tendon [63]. A meta-analysis [23] supports the use of EE for AT, and additional benefits can be produced when EE is used in conjunction with laser therapy.

Eccentric exercises have been tested against concentric exercise; rest/wait-and-see; light training; heavy slow resistance; or another eccentric exercise protocol. There is a wide variation between the trials regarding interventions, methods, times of evaluation of the results, and selection of the reported results.

One study investigated EE and concentric exercise. This study [48] found improvement in pain and function/return to activity in both groups, with the EE producing significantly better results. Mafi et al. [48] suggested that EE produced significantly better results than concentric exercises because of the energy efficiency of eccentric exercises on the calf muscles, with comparable muscle force developed at lower loads on the tendon during movement. In addition, EE may produce changes in the metabolism of neural transmission in the tendon, inducing alterations in the perception of pain. This corroborates Yu et al.’s [46] report that EE is more effective than concentric exercises in reducing pain, increasing muscle strength and endurance, and improving function. However, the small sample does not allow generalization of the results [64].

Two studies compared EE with rest/wait-and-see. Rompe et al. [51] verified improvement in pain and function of the EE group, which, despite not presenting a statistical difference, showed considerably better results than the control group. They report a successful outcome in the EE group of 50 to 60% of patients, reporting that eccentric training is inexpensive, although it is technique dependent. The wait-and-see protocol was the most convenient and easy intervention to implement, but also the least effective [51]. Horstmann et al. [52] showed significant improvement in the EE group compared to the control group, in addition to a significant reduction in pain on palpation of the EE group. While interventions improved pain two cm proximal to the insertion of the Achilles tendon in the calcaneus, only EE reduced pain at the musculotendinous junction as well. They reported that pain reductions following eccentric training correlated with reduction in neovascularization in patients with tendinopathy, although such changes in vascularization were not actually observed.

As in several studies with active versus rest/wait-and-see treatment, individuals receiving active treatment may have higher expectations about the effects of treatment, and testing different intensity exercises against a rest/wait- and-see can lead to bias in the conclusions [65, 66].

Silbernagel et al. [49] compared light training (eccentric plus concentric) and EE, and verified improvement in palpation, walking and activity pain, as well as improvement in functional tests and ROM for the EE group, with no statistically significant difference between the groups. The reason for improvement in the experimental group may be multifactorial, in addition to being explained by the different components of the treatment protocol used by the experimental group, such as the information provided, the exercise program, and the pain monitoring model. The authors also report that the exercise load has to be relatively high for better results [49]. Beyer et al. [55] presented a comparison of the EE and HSR groups. Both interventions showed significant gains in improving physical activity and pain, but without statistical difference between groups: the treatments are similar to each other, although patient satisfaction tended to be higher after 12 weeks with HSR (100%) than EE (80%). This may explain why these two studies [49, 55] are more towards the right side of the forest graph. Silbernagel et al. [49] further state that the experimental group had an increase in pain in the first weeks of treatment, and that this may resulted from the increased load. Both treatments improved symptoms and physical activity level equally well in patients with chronic midportion AT [55]. Eccentric and concentric contractions produce similar collagen expression, which may indicate that fibroblasts are similarly affected. Furthermore, training with concentric and eccentric contractions can produce similar tendon changes. The authors state that they cannot answer the question regarding the mode of contraction, but both treatments promoted similar results. Kongsgaard et al. [67] compared EE and HSR for patellar tendinopathy, with similar results between the interventions: it is possible that the combination of eccentric and concentric exercise may explain the advantageous effects of HSR [67].

Three studies compared EE with EE. The first [50] applied the same treatment protocol, but only one group was released for physical activity. Both groups presented improvement in pain and function during the evaluations, with no statistically significant differences between the groups. The study demonstrated no negative effects in patients who continued physical activity (such as running and jumping) when using pain monitoring during rehabilitation and believe that important factors in tendon improvement are intensity and type of load. The underlying effects of exercise are not fully understood, but the mechanical load on the tendons seems to be important both in the healing process and in the increase in tendon strength. The second study [53] compared Alfredson’s and Stanish’s protocols. The former reduced pain and improved function to a greater extent than the latter. The protocol developed by Alfredson et al. [32] is a program of eccentric exercises to treat the AT, while the uninjured limb is used to return (concentrically) to the initial position. The protocol recommends the completion of 180 eccentric repetitions per day, and has been widely adopted in research and clinical practice [32, 40]. Stanish et al.’s [68] protocol for the management of AT includes eccentric and static stretching exercises and is based on three principles. (1) length; (2) load; and (3) contraction velocity. According to the study authors [53], the protocol by Alfredson et al. [32] reduced pain and improved function more efficiently because patients exercised both calf muscles (gastrocnemius and soleus) only eccentrically, with more series and with more repetitions every day for the same treatment period [53]. In addition, the load of EE in the Alfredson’s protocol was increased according to the patients' symptoms, and the exercises were performed at low speed, which is supposed to allow suitable tissue adaptation [53].

The third study applied only the Alfredson protocol [54]. One group was asked to perform 180 repetitions, and the other performed until tolerated. A statistically significant difference was found for improvement in function in each group at three weeks and for pain in the “do-as-tolerated” group; statistically significant differences between the groups in improvement of function were evident at week three, but by six weeks there was no statistical differences between the groups for pain and function. Regarding the outcomes, given the limits of the scoring systems used, the "do-as-tolerated" regime can equal or even exceed the standard protocol. In addition, defining a dosage may be important for rehabilitation, suggesting that a clinical predictor based on worsening symptoms may be used by patients who have demonstrated optimal clinical improvements. This may be a potential benefit to improve self-efficacy, which has been associated with positive results for the treatment of musculoskeletal conditions [54]. However, as the exercises were only performed for a period of 6 weeks, and the follow-up measurements in the medium and long term (> 6 weeks) were not presented, these conclusions should be interpreted with caution.

This systematic review and meta-analysis focused only on studies comparing EE with another type of exercise or control group (rest/wait-and-see). One of the most interesting findings lies on the favourable results produced by this type of exercise, suggesting that EE should be an integral component of AT management. Also, the rest/wait-and-see approaches do not provide any significant benefits when compared to EE. In addition, controlled tendon loading can continue during the intervention, though further studies are needed to determine which activities are beneficial, and their frequency and intensity are recommended.

The present review has identified several characteristics that should be adopted for future clinical trials. With "some concerns" and "high risk" through the risk of bias, new randomized clinical trials should be performed and adhere to the recommendations of CONSORT [69]. In addition, specific, valid and reliable outcome measures should be used [15], facilitating comparisons between different studies and when performing systematic reviews and meta-analyses.

We are aware of the limitation of the present investigation. Only one meta-analysis was performed, namely on pain, since it was not possible to group more than two studies for disability analysis. It is necessary to collate a larger number of studies to achieve greater statistical power [70]. In addition, there is a substantial need for studies with larger sample size, greater details of the sample and the proposed interventions, as well as better methodological modalities and a planned design. Another limitation would be that half of the selected articles presented at least some risk of bias in relation to randomization. Ultimately, most of the articles analysed present some concerns such as heterogeneity of the study population, and lack of reporting of training compliance data or high risk of bias.

## Conclusion

The available evidence supports the use of EE in the management of AT. Continuous load on the Achilles tendon does not adversely affect the results of pain and function, suggesting the possibility of practising some physical activities during the intervention. Some authors report that EE and HSR produce similar results, but more studies are needed to confirm this. Also, the rest/wait-and-see approaches probably do not play a role in the management of AT.

## Data Availability

All data generated or analysed during this study are included in this published article.

## Abbreviations

ROM: Range of motion
AT: Achilles tendinopathy
EE: Eccentric exercise
RoB2: Risk of Bias 2
MD: Mean difference
CI: Confidence interval
HSR: Heavy slow resistance
